# An unusual presentation of autosomal dominant polycystic kidney disease in a newborn

**DOI:** 10.11604/pamj.2024.48.146.44523

**Published:** 2024-08-02

**Authors:** Aditi Rawat, Mahaveer Singh Lakra

**Affiliations:** 1 Department of Neonatology Jawaharlal Nehru Medical College, Datta Meghe Institute of Higher Education Research, Sawangi Meghe, Wardha, Maharashtra, India,; 2 Department of Pediatrics, Jawaharlal Nehru Medical College, Datta Meghe Institute of Higher Education Research, Sawangi Meghe, Wardha, Maharashtra, India

**Keywords:** Neonatology, nephrology, polycystic kidney disease

## Image in medicine

A 2.3 kg female newborn born to a primigravida with no history of consanguinity at 36 weeks of gestation, by cesarean section for preterm labor. Antenatal history was suggestive of oligohydramnios with an amniotic fluid index of 7 and bilaterally enlarged fetal kidneys. No history of renal disorders is present in the family. The baby cried immediately after birth. On examination, vitals were stable without any respiratory distress. Abdominal examination revealed a massively enlarged abdomen with visible dilated veins (A) and bilateral ballotable masses suggesting renomegaly. There were no facial or body dysmorphisms for syndromic association. The blood pressure was within the normal range. Ultrasound abdomen showed enlarged hyperechoic kidneys with multiple cysts throughout the renal parenchyma (B). No cysts were seen in the liver and spleen. The baby was passing urine adequately. The renal function test showed serum urea of 45mg/dl serum creatinine of 1.5mg/dl at 48 hours of life, serum sodium of 138 meq/L, and serum potassium of 4.7 meq/L. Genetic testing confirmed biallelic mutation in the PKD1 gene on chromosome 16. A final diagnosis of autosomal dominant polycystic kidney disease (ADPKD) was made. As there were no clinical symptoms like oliguria, respiratory insufficiency, or hypertension, the baby was discharged on the 7^th^ day of life. Regular follow-up was explained for monitoring renal functions and hypertension as there is an 8% and 15% risk of chronic kidney disease and hypertension respectively by adolescence. Usually, the presentation is in adulthood but this unique case has a rare prenatal presentation despite being of autosomal dominant variety.

**Figure 1:**
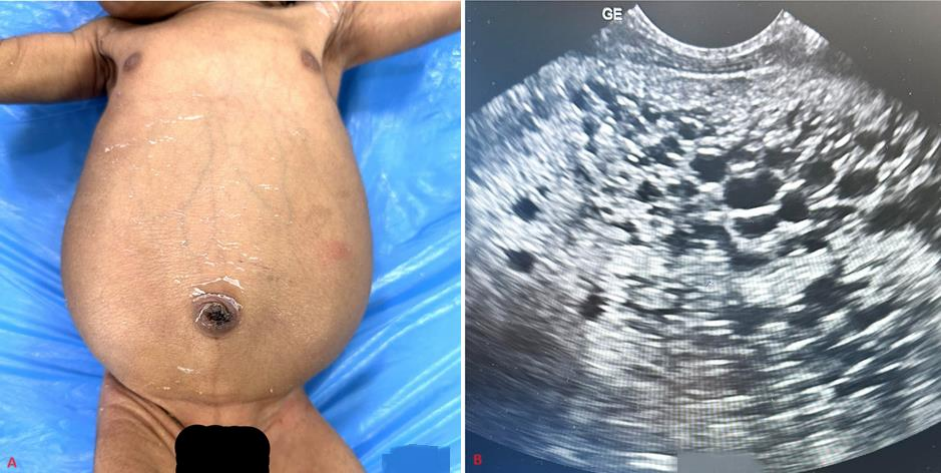
polycystic kidney disease with: A) abdominal distension; B) multiple cysts in the renal parenchyma on ultrasonography

